# Original Translations

**Published:** 1882-07

**Authors:** 


### Original Translations.

DEMOKEDES, OF KROTON.—(*Archiv für Gerschichte der Medicin.*)

At Knidos there lived an Æsculapian priest, with the name Kalliphon, who was very well educated in the art of medicine. To him was born a son, and he called him Demokedes. When the youth was growing in limbs the father tried to cultivate his brain, and taught him, beside classic matters, the highly esteemed art of medicine. When his son was grown up they removed to Kroton, as the river was named, and practiced their profession. But they could not agree, the father being a rather passionate man, but the son a very gentle youth. Therefore, Demokedes migrated to Ægina, where he surpassed even in the first year all his old colleagues. At this time (about 500 B. C.,) a physician had to do not only general practice, but had also to mix his medicaments, sew his bandages, and to manufacture all his instruments. Young Demokedes, notwithstanding that he had no appliances whatever, excelled by his practical ways in so marvelous a manner that he was appointed after the first year as city physician, with $1600 salary, the first appointment ever made of this kind.

After being three years at Ægina, his fame was so widespread that he was called as city physician to Athens, with a salary of $2600, but after a little while the famous Prince of Samos, Polycrates, made him a court physician, with $3200. Here he lived in a princely style as the highly esteemed friend of the Prince of Samos. But life is changeable. Orœtas, Persian Governor of Sardis, was a mortal enemy to Polycrates from unknown causes, and in a deceitful manner he invited the Prince

Polycrates to visit him, with all his court followers, at Magnesia, where the unlucky prince was captured and killed. Our Demokedes, who had accompanied the prince, was sent as a slave to Darius, King of Persia (485 B. C.). Here the unhappy Demokedes lay among other slaves, clad in rags, with chained hands and feet for a long time, but a happy accident raised him again to the highest honors. Darius, the mighty king, jumped one day from his haughty stallion (or was thrown off), and got a luxation of his ankle-joint. The Arabian physicians of the court were unable to reduce the luxation, and inflammation set in. When the king lay in great agony, and his physicians were unable to do anything, somebody told him of Demokedes. Clad in his rags the latter was brought before the king, and asked if he were a physician. "No;" said our Demokedes, who was afraid that he would never again see the borders of happy Hellas, but when he was threatened with torture he agreed to help the king. And so he did. The king, after being restored to full health, presented two chains of pure gold to Demokedes, which the latter refused, asking the king if he would chain him forever. Then the king sent him to the dames of his seraglio, and they filled a large bucket full with gold, so that the servant of Demokedes, who picked up the overflowing gold pieces, made quite a large fortune. He was then elected court physician, and lived again in a princely style as the most esteemed friend of the mighty King Darius. But he always longed to see his happy Hellas again.

Among his patients was Atossa, daughter of the King Cyrus, II, and wife of Darius. She had got a small sore on her breast, which she kept secret in false bashfulness as long as possible, until it developed to a regular mastitis. Then she called Demokedes, and he promised to cure her if she would do what he asked from her for his benefit and not to her own danger. So she said to Darius: "O mighty king, you are such a strong and powerful young man, and you have so many soldiers who have nothing to do but eat and drink; can you not go and make war upon the Greeks and bring me some of those beautiful Greek maid servants?" "That I will do," said Darius, and he ordered fifteen commanders of his army to go to Greece and look at their armies and at their fortified cities. But Demokedes was elected as the leader. Two war vessels were equipped and a large ship laden with costly presents to the brothers of Demokedes was added. They sailed to Greece, and there Demokedes took his departure with the costly presents and bade the commanders good-bye. They tried to capture him, but the citizens of his native city Kroton defended him. Afterward he sent to the King Darius his card of marriage with the daughter of the celebrated Milo. At Kroton he started a medical school, and he was by far the most celebrated physician in Greece after Hipocrates, and to his college came all the young men from over the world, who would study the art of medicine.

THE ETIOLOGY OF TUBERCULOSIS.—(*Wien. Med. Chir. Centralbl.*)—Villemin was the first to discover the conferring of tuberculosis upon animals. The inoculation of tuberculous matter into animals has been tried by Cohnheim, Solomonson and Baumgarten, and Tappeiner proved the possibility of conferring the disease by inhalation. The statistical tables show that one-seventh part of all human beings die from tuberculosis, and if we take the lower classes alone, one-third are destroyed by this terrible disease. Koch, the medical counsellor of the German empire, has investigated the matter thoroughly. The experiments made on this subject had been hitherto without success, because the methods of dyeing the micro-organisms had not been satisfactory, and even Cohnheim, the first investigator, says in his recent book on general pathology, that the direct proof of tuberculous virus is a problem not yet solved. Dr. Koch has dyed the virus in the following manner: The objects to be examined are dried on the objective glasses. They are then put in a solution of 200 ctm. of distilled water, in which is dissolved one ctm. of concentrated solution of methylene blue. To this is added a 0.2 ctm. of a 10 per cent. solution of caustic potash. The glasses are kept there for twenty-four hours, and then they are put in a concentrated aqueous solution of vesuvine and cleaned with distilled water. By this process all the other tissues take on a brownish color, but the bacilli are stained blue. The bacilli thus prepared show a peculiar appearance. They have a rod-like form, are very thin, and one-quarter of the diameter of red blood corpuscles long. On those points where the tuberculous process is in rapid progression, the bacilli will be found in densely packed groups and bunch-like aggregations. (In all the hospitals at Berlin the diagnosis of tubercles in difficult cases is now made in this manner by the microscope.)

St. Petersburg.—Surgeon-General Busch, of the navy, has been sentenced to four years' banishment to Siberia and discharge from the navy for "selling offices." Happy Russia!

Impotentia Viris.—(*Gazetta Ukarska*).—A healthy man, twenty-four years of age, who practiced masturbation for a certain time, was sued by his wife for impotence, and she got a divorce. From this time on the man suffered from attacks of melancholia, but recovered and paid his addresses to another woman. A short time before the stipulated marriage he had the same attacks, and he became so affected that he tried to commit suicide. Dr. Janiszewski found the patient to be well developed with but a slight sensibility on the lower dorsal vertebræ. The prepuce of the penis had a low temperature. The doctor thought, in absence of any pathological symptoms, the case to be of a moral character, and treated the patient accordingly. He persuaded him to marry, but he forbade cohabitation for the first two weeks. At the same time he galvanized the patient, one electrode applied to the dorsal vertebræ, the other to the scrotum. Cold baths were administered to the lower part of the body and small doses of strychnine were given. After the time named the patient recovered.

Sudden Death in Phthisis Caused by Entrance of Air into the Blood Vessels.—(*Berl. Klin. Wochenschr.*)—A patient twenty-two years old, of a phthisical family, showed indistinct respiration with dullness on the right lobe of the lung. The expectorated matter was greenish and feculent. After being three weeks in the hospital he suddenly collapsed and died without convulsions. The autopsy showed tubercles in the right lobe and a small cavity in the upper part of the same lobe. There were three small cavities in the left lobe, two being filled with caseous matter, the third with blood and air. The heart was somewhat enlarged, and showed the left ventricle filled with blood and air, as was also the right ventricle. All the main arteries contained blood with air bubbles. All the arteries and veins of the brain were filled with air. The air in the chest had broken into a branch of the pulmonary vein, and thus by the action of the left ventricle the air was forced into the whole system.

LA FRANCE MEDICALE says that the radical cure of hydrophobia as reported by Bonley and Dartigue, by means of *hypodermic injections of pilocarpine* is not so reliable as thought at first. Dr. Dartigue admits that he administered at the same time arsen. of strychnine, hyoscyamine and monobromate of camphor. It is difficult to distinguish the effects of this "mitraille" of medicaments.

### HYSTERIA IN THE MALE.

It is now admitted by all practitioners, that hysteria is not exclusively the bane of women. Man may also, in some cases, present all the characteristic features of this disease: vomiting; epileptiform crises; paralysis; choreic movements; anorexia; nosomania (mimicry of diseases); necrophobia; all of which may appear in individuals of the male gender with the same intensity and the same intractability as in the female, or be relieved as promptly, under the same treatment.

While perusing the proofs of a concise treatise on Hydrotherapy (now being published), through the kindness of Mr. Pascal, we have been surprised by the number of adolescents who have applied to the Hydrotherapic Institute of Passy, to be cured of this disease. And what is remarkable, is that a number of these youthful patients came from the most distant and different countries. Whether Russians, English, French or Swiss, their symptoms were always identical. (In his work on hysteria major, Mr. Paul Richer made the same remark in regard to women.)

Here are, in a few words, some of the cases which will attract practitioners' attention in the *Précis d'Hydrothérapie.* just referred to :

The first case is that of a young man from Nancy, emaciated, and affected from his infancy with marasmus. When presented to Prof. Charcot, he was suffering from vomiting and paraplegia, and placed under the care of a parent who allowed him everything that he wished. Brought to Passy, this case was treated by the water-cure; two douches a day; temperature 11° C. After a month, he had regained the power to walk, and he was put on gymnastics. A month later he began to learn fencing. Three months from the time of his admittance, he left the establishment entirely transformed, and as vigorous as the boys of his age.

Another boy from Nancy, æt. sixteen, came to Passy after trying all the ordinary means of cure. His parents had neglected nothing that was of a nature to relieve him. In despair of any success, they applied to Prof. Charcot, for whom a diagnosis was easy. His nervous crises and paraplegia could be nothing else but hysterical manifestations. This young patient was put under the care of a professor whose duty it was to give him intellectual work to do in the institute. While under treatment he had a relapse, following a five weeks' sojourn in his family. Patient afterward left the establishment cured.

Another case just as remarkable was that of a boy from Dunkerque, æt. eleven. In 1875 he had applied to Dr. Barthez, who, seeing the long standing of the disease, the number of physicians employed, and the variety of therapeutical means used, called Prof. Charcot in consultation. Both concurred in making a diagnosis of hysteria, and recommended rational hydrotherapy. In this case likewise, paraplegia was a predominating symptom. The leanness was extreme. There was anorexia in the way of alimentation. After a month's treatment, appetite had returned, and the youth could jump on one leg. After two months, he could walk naturally. After three months he left the institute, in spite of the doctor's advice to the contrary. Five months later there was a relapse. A new course of hydrotherapy cured the patient definitively.

Among other cases, Mr. Pascal cites that of a boy ten years old, from Dieppe, who was treated in Passy about the time of the preceding patients. Anorexia and paraplegia were again the main symptoms. The same treatment was used ; two douches a day, temperature 11° C., filiform douche; later, gymnastics; during the whole treatment, a severe discipline. Same success; that is, a cure in two months.

A boy twelve years of age, from Paris, was another instance. He was an only son. He was under the care of a well-known physician of the capital, but his parents, frightened at the doctor's diagnosis of a cerebral tumor, consulted Charcot, who diagnosed hysteria. The position in a circular arc during a crisis, and the craving of the boy for dolls, and all things looked after by little girls, were evident symptoms. He was sent to the hydrotherapeutic establishment, and the cerebral tumor disappeared under the repeated use twice a day of the spray, and the filiform douche. The treatment was continued for five months. The last case was that of a tall child, son of a nervous mother, who had, beside paraplegia, spasms of the œsophagus. He was unable to feed himself, and was alimented through a sound. After ten days' treatment, he was able to feed in the way of other people, and six weeks from the time of his arrival at Passy, he returned to his family perfectly well.

This brings us to the conclusion that the organization of water-cures in all our hospitals commends itself to Boards of Health (*Assistance publique*) in every city in France, and that the adoption of Fleury's doctrine by all who make use of baths in their treatment, will prove a great progress in the therapeutics of nervous diseases, so common in this age, and so discouraging for those who have not made a special study of hydrotherapy.—*L' Union Médicale.*

HEREDITY. — Brown-Séquard, at a meeting of the French Academy of Sciences, March 13, 1882, presented a communication on experiments made on Guinea-pigs, which prove that accidental affections of the parent are sometimes transmitted to the offspring.—*Archiv. Gen. de Médicine.*

ARTIFICIAL RESPIRATION.—Dr. De Chilly presented the following method of treating asphyxia: There is a mechanical means, extremely facile in its application, to force the asphyxiated to breathe with full lungs. It consists in converting the chest into bellows, the sides of which are represented by the ribs. In order to effect this result, it suffices for the subject to lie on the back, and as much as possible, on an inclined plane. The operator, standing on one side, with his face turned toward the subject's feet, dips the four flexed fingers of each hand as high as possible under the false ribs on either side, and grabs them firmly. This prehension should be made on either side where the parietes of the chest project the most below. Now, the operator executes alternate motions, elevating and depressing the ribs which give the chest its greatest expansion and compression, for the motions affect the whole sides of the chest.—*Bulletin de l'Académie de Médecine.*

This process is not new, but the description will refresh some readers' memories.

TREATMENT OF WHOOPING COUGH.—Jules Simon acknowledges that no specific exists for this disease, and after trying all the modes of treatment which have been extolled in turn, he falls back on the use of tr. of belladonna and tr. of aconite root in equal parts, giving two drops of the mixture morning and evening, and increasing to five or ten drops, according to age. After attaining the large doses, he diminishes them gradually to the original dose. At the same time, two or three emetics are given every week. For a child two years old, a teaspoonful of the following mixture will do:

| | | |
|---|---|---|
| ℞ Powd. ipecac........................... | .0 | 5 |
| Syr. of ipecac........................... | 30 | |
| M. | | |

Should the child vomit his food during a crisis, he should take a dessertspoonful of black coffee, and eat another meal.

The main indications to fill are, above all to avoid cold, and observe the strictest hygienic precautions. The child should remain ten days in bed, and stay indoors for a month or six weeks, according to the season of the year.—*Le Praticien.*

TREATMENT OF APHONIA IN SINGERS AND ORATORS.—Dr. Corson, in the *Revue Médicale*, advises in this trouble to take a piece of borax of .15 or .20 grams into the mouth and let it melt. An abundant secretion of saliva takes place. Public speakers, lawyers, preachers, etc., should take, the day preceding that when they have to speak, a glass of sweetened water in which is dissolved 15 grams of nitrate of potassium, which will cause profuse perspiration. Under the same circumstances the following gargle will be useful:

| | | |
|---|---|---|
| ℞ | Alum | 5 to 10 |
| | Honey | 20 |
| | Barley decoction | 200 |
| | M. | |

Another means consists in the use of an infusion of jaborandi, three grams in a cup of boiling water, to be taken in bed in the morning, to cause a good perspiration.—*Lyon Médical.*

EXPERIMENTAL RESEARCHES ON THE GRAFTING OF DEAD BONE UPON LIVING BONE; RESORPTION OF SEQUESTRA.—Mr. Lannelongue recently made a communication on this subject before the Surgical Society, May 17, 1882. Says the doctor: "A true sequestrum may exist under two conditions: it may either be bathed in pus, or be surrounded with granular membranes whose granulations come in contact with its sides and sinuosities. In the first case, clinical observations and experiments have shown that a sequestrum remains indefinitely *in statu quo*. In cases where granulations exist, there may be a good deal of absorption." Experiments made by Billroth on the process of resorption of ivory implanted into living bones or tissues, together with those of Langenbeck and Diffenbach, have supported that opinion. But Mr. Lannelongue's experiments were to the point. He used fragments of bone found in the halls of amphitheaters, where they had laid for years, and, after previous disinfection, introduced them into the bones of animals, in which they were subsequently absorbed. This is a demonstration that sequestra may, under some conditions, be reabsorbed.—*L' Union Médicale.*

